# New PCR-specific markers for pollen fertility restoration *QRfp-4R* in rye (*Secale cereale* L.) with Pampa sterilizing cytoplasm

**DOI:** 10.1007/s13353-021-00646-z

**Published:** 2021-06-25

**Authors:** Agnieszka Niedziela, Marzena Wojciechowska, Piotr Tomasz Bednarek

**Affiliations:** Department of Plant Biochemistry and Physiology, Plant Breeding and Acclimatization Institute – NRI, 05-870 Błonie, Radzików, Poland

**Keywords:** Rye, Cytoplasmic male sterility (CMS) Pampa, Pollen fertility restoration, Molecular markers

## Abstract

Pampa cytoplasmic male sterility phenomenon is used extensively in the rye hybrid breeding programs. It relies on sterilizing action of the cytoplasm resulting in non-viable pollen of female lines. The sterilizing effect is problematic for reversion, and efficient restores are needed. The most promising QTL is located on chromosome 4R, but other chromosomes may also code the trait. Advanced recombinant inbred lines formed bi-parental mapping population genotyped with DArTseq markers. Genetic mapping allowed the seven linkage groups to construct with numerous markers and represent all rye chromosomes. Single marker analysis and composite interval mapping were conducted to identify markers linked to the pollen fertility. Association mapping was used to detect additional markers associated with the trait. A highly significant QTL (*QRfp-4R*) that explained 42.3% of the phenotypic variation was mapped to the distal part of the long arm of the 4R chromosome. The markers localized in the *QRfp-4R* region achieve R^2^ association values up to 0.59. The homology of the 43 marker sequences to the loci responsible for fertility restoration in other species and transcription termination factor (mTERF) linked to *Rf* genes was established. Ten markers were successfully converted into PCR-specific conditions, and their segregation pattern was identical to that of unconverted DArTs.

**Supplementary Information:**

The online version contains supplementary material available at 10.1007/s13353-021-00646-z.

## Introduction

Cultivated rye (*Secale cereale*) is a cross-pollinated, diploid plant species known for its high tolerance to low winter temperatures and better withstanding adverse soil conditions than other cereals. Its cultivation area is focused mainly in the north-eastern and central parts of Europe, Poland, Germany, and the Russian Federation, which deliver over 58% of world grain annually.

The rye breeding programs focus on improving the high seed yield achieved via hybrid breeding based on heterosis. The exploitation of the heterosis effect results in a 20–25% higher grain yield than population breeding using the same agriculture (Geiger and Miedaner 1996; Hansen et al. 2004; Laidig et al. 2017). Hybrids' success depends on selecting inbred parental lines with the superior combining ability and exploiting the cytoplasmic male sterility (CMS) phenomenon. Cytoplasmic pollen sterility relies on the mitochondrial genome dysfunction that prevents the maturation of the male sex organs (stamens), resulting in defective pollen or its lack. Therefore, the mating system in rye hybrid breeding requires three components, a maternal line carrying the CMS allele, a non-restorer germplasm for maintaining the CMS and a paternal line carrying restorer-of-fertility (*Rf*) nuclear genes which are able to reverse CMS effect.

Distinct cytoplasms have been described in the rye and classified into two groups based on their restoration ability. The easily restored sterilizing cytoplasms are represented by Vavilovii (CMS-V), CMS-R (Kobyljanskij 1969), CMS-C (Łapiński 1972), and CMS-G (Adolf and Winkel 1985), and others, whereas Pampa (CMS-P) cytoplasm is the only representative of a hardly restored source. Among soft CMS only G source was used for the production of hybrid cultivars. The example here is the “Novus” cultivar registered in Germany in 2000 (Melz and Adolf 1991; Melz et al. 2001). Two other cultivars based on the G cytoplasm, “Hellvus” and “Helltop” are no longer exploited. It seems that one of the potentially relevant sources of CMS in rye could be the C cytoplasm (CMS-C) discovered by Łapiński (1972) in the old Polish cultivar “Smolickie.”

The CMS-P, characterized by a robust sterilizing effect, is widely explored in hybrid breeding since the 70’s (Geiger and Schnell 1970; Geiger and Miedaner 1996). It was found that restoration of pollen fertility in rye with CMS-P depends on QTLs mapped to the 1R, 3R, 4R, 5R, and 6R chromosomes (Miedaner et al. 2000). In European rye resources, the strong QTL explaining 54% of the phenotypic variation was detected in the German inbred line L18 on chromosome 1RS (Miedaner et al. 2000). The other helpful fertility restoration QTL (*Rfp2*) was mapped to the long arm of chromosome 4R in the Argentinian cultivar “Pico Gentario” and Iranian primitive rye accessions called IRAN IX (*Rfp1*) and Altevogt 14160 (*Rfp3*) (Geiger and Miedaner 1996). The Rfp QTLs in the European populations are rare (1–5%) (Geiger et al. 1995) but were successfully introgressed into elite pollinator germplasm from Iranian resources.

The precise mechanism of pollen fertility restoration and the role of genes involved in this process have not been assessed until now. In most tested species, the *Rf* genes encode mitochondria-targeted pentatricopeptide repeat (PPR) proteins (Zhao et al. 2018; Wang et al. 2006; Akagi et al. 2004; Brown et al. 2003; Desloire et al. 2003; Klein et al. 2005; Fujii et al. 2016; Melonek et al. 2019; Goryunov et al. 2019). They play a pivotal role in organelle RNA editing, RNA termination, splicing, and translation (Hammani and Giegé 2014). Most of the *Rf* encoding PPR proteins (Rf-PPR) belong to the P class repressing ORFs. The latter causes mitochondrial dysfunction and pollen abortion in flowering plants (Dahan and Mireau 2013; Kubo et al. 2020). Further, sorghum *Rf*1 (Klein et al. 2005) and barley *Rfm*1 (Rizzolatti et al. 2017, Melonek et al. 2019) are classified into the DYW subclass in the PLS subfamily (Lurin et al., 2004). These types of PPR have been associated with C-to-U RNA editing. They thus could prevent the accumulation of the sterility factor by either creating a stop codon within the CMS-inducing transcript or an amino acid substitution in the putative sterility protein (Rizzolatti et al. 2017). Still, to date, such a mechanism has not been supported by experimental data. The other candidate gene implicated in fertility restoration belongs to the mitochondrial transcription termination factor family (mTERF). The mTERF was closely linked to *Rfp1* and *Rfp3* in the rye (Hackauf et al. 2012, 2017) and *Rfm3* in barley (Bernhard et al. 2019); however, its function in the context of fertility restoration has yet not been reported.

A high linkage drag of undesirable genes with the 4R QTL was observed in hybrids (Miedaner et al. 2000, 2017). Two markers, namely Xtc256739 and Xtc300731, flanking the *Rfp1* gene at distances of 0.3 cM and 0.4 cM were evaluated (Hackauf et al. 2012). However, such a linkage was insufficient to eliminate the drag effect, and newly developed, more linked markers are not readily available. To identify such markers, either the F2 mapping populations based on CMS Pampa cytoplasm are required, or recombinant inbred line (RIL) mapping population is needed. Vast mapping populations are needed to achieve a high resolution of genetic maps based on the F2 progeny. Their genotyping is expensive if dense maps are needed as large populations need to be genotyped. The other option relies on advanced recombinant inbred lines. However, their evaluation may be problematic due to the inbreeding effect in the rye. Nevertheless, the employment of RILs, if available, should decrease genotyping and phenotyping costs.

The study aims to identify pollen fertility restoration QTLs in rye hybrids with the CMS Pampa cytoplasm. Furthermore, we were interested in identifying genes responsible for pollen fertility restoration. Finally, we converted markers for marker-assisted selection purposes.

## Material and methods

### Plant materials and phenotypic evaluation

The rye mapping population S60/08 encompassing 94 recombinant inbred lines (RIL F8 (S60/08): S 305 N/00 × SO 2R/05) was derived in Choryń by crossing the S305N/00 (maintainer) plant on non-sterilizing cytoplasm and male parent SO2R/05 with CMS Pampa (restorer line). The single-seed descents method allowed the development of the RIL mapping population up to the F8 generation. The test crosses between the sterile female source of CMS Pampa (S305P/00) and a given recombinant inbred line allow obtaining BC1F1 (S305P/00 × [RIL F8 (S60/08): S 305 N/00 × SO 2R/05]) materials. From 10 to 12 plants for each of the 94 cross combinations were grown during the vegetation season 2018/2019 in the field conditions. The average value of fertility/sterility based on ten plants of BC1F1 was assessed according to the bonitation scale proposed by Geiger and Morgenstern (1975). Extremely male-sterile plants were scored as 1, whereas plants with anthers that intensively released pollen were scored as 9. Partly fertile plants exhibited values in the 4–6 range.

The pollen fertility restoration trait normal distribution was tested using the Kolmogorov–Smirnov test implemented in XlStat software (XlStat 2019). The *χ*^2^ goodness-of-fit test was conducted to determine whether the data “fit” the expected 1:1 distribution using MapQTL 5 (Van Ooijen 2004).

### DNA extraction

Genomic DNA was extracted from fresh leaf tissue of parental plants, and a single individual represented each of the 94 RILs (F8) using a DNeasy Plant Mini Kit 250 according to the manufacturer’s instructions. DNA concentration and purity were checked using the PicoDrop spectrophotometer. The integrity was assessed by electrophoresis, applying about 100 ng DNA on 1% agarose gels stained with EtBr (0.5 µg/ml) in TBE buffer.

### Genotyping

DNA samples were sent to Diversity Arrays Technology (Pty) Ltd. in Canberra, Australia for genotyping using DArTseq™ technology, which is a combination of the DArT complexity reduction method (Jaccoud et al. 2001) and Next-Generation Sequencing (NGS) platform. The DArTseq protocol has been described in detail by Kilian et al. (2012) and Melville et al. (2017). It encompasses the following steps: (1) Digestion of DNA samples using the restriction enzymes (RE) combination most suitable for genome complexity reduction—at least one of the enzymes is methylation-sensitive, directing the analysis to the hypomethylated, gene-rich genome regions; (2) Ligation of specialized adaptors to the digested DNA; (3) Amplification of adaptor-ligated fragments by adapter-mediated polymerase chain reaction (PCR); (4) Sequencing of the amplification product for each sample on an Illumina HiSeq2500 (Illumina Inc., San Diego, USA).

The results consist of two groups of detected markers based on short DNA sequences. The first group is called SilicoDArT and represents markers classified as dominant and scored for the presence or absence of a single allele. Such markers arise due to the presence or absence of restriction sites. The second group encompasses codominant single nucleotide polymorphisms (SNPs) markers carrying a single nucleotide polymorphism identified within marker sequence. As our materials were highly homozygous, both SNP and SilicoDArT markers were coded as dominant markers in a 0–1 binary form.

### Linkage map construction

The genetic map was constructed using MultiPoint Ultra-Dense software (Ronin et al. 2015). Markers exhibiting > 15% missing data were excluded. All SNP and silicoDArT loci that showed no or minimal deviation from the expected 1:1 segregation ratio (*χ*^2^ ≤ 19.2) were employed in the analysis.

Genetic map construction consisted of the following steps: (1) Markers with zero distance were grouped, and a “delegate” was selected from each group. Only markers with at least the same number of twins as the predefined threshold were selected as delegates and were defined as "skeleton." Markers exhibiting identical segregation patterns as the delegate/skeleton markers were assumed redundant; (2) All remaining markers, except for candidate twins, were removed to the Heap; (3) Most representative skeletons markers and their redundant counterparts were clustered, and the resultant linkage groups (LGs) were ordered; (4) Gaps were filled, and LG ends were extended using markers from the Heap (Heap contains markers due to, i.e., segregation problems or missings primarily removed from mapping procedure). Such markers were referred to as approximated to the map; (5) Markers violating map stability and monotonic growth of distance from a marker and its subsequent neighbors were removed.

### Quantitative trait loci (QTL) analysis

Single marker analysis (SMA) using the Kruskal–Wallis rank-sum test (P < 0.005) in MapQTL version 5.0 (Van Ooijen 2004) and the composite interval mapping (CIM) procedure in Windows QTLCartographer software, version 2.5 (Wang et al. 2007) was performed to identify QTLs responsible for fertility restoration in the S60/08 population of rye. A backward regression method with a window size of 5 cM and a walking speed of 1 cM, with control markers equal to five, was used for CIM. The logarithm of the odds (LOD) thresholds for significance was obtained by MapQTL’s permutation test option (1000 permutations). The percentage of phenotypic variation explained by each QTL was estimated. The QTL mapping data are provided in Supplementary File S1.

Verification of the marker’s order for linkage groups with detected QTLs was performed using a high-density genetic map based on rye Lo7xLo225 mapping population and anchoring Lo7 WGS contigs (Bauer et al. 2017). For this purpose, a homology between marker sequences localized on the individual linkage groups of S60/08 and sequences of contigs localized on the individual rye chromosomes of the Lo7xLo225 genetic map was searched. Pearson’s correlation in Statistica ver. 13.3 was used to verify the extent to which the marker’s order of the two maps was comparable (TIBCO Software Inc. 2017).

### Association mapping

Principal Coordinates Analysis (PCoA) was performed in PAST software to assess the genetic structure of the RILs forming a mapping population. Associations of SNP and silicoDArT markers with pollen fertility restoration were determined using a General Linear Model (GLM) in TASSEL version 3.0 (Bradbury et al. 2007). Significant associations were indicated by the Bonferroni test with p < 0.01 (0.01/number of markers). The degree of association was illustrated by the determination coefficient (R^2^). Marker’s position on the S60/08 genetic map and the high-density genetic map constructed by Bauer et al. (2017) were determined.

### Bioinformatic analysis of the marker sequence homology

Sequence similarity search algorithm BLASTn available for use online at the National Center for Biotechnology Information (NCBI) website (https://blast.ncbi.nlm.nih.gov) were used to generate alignments between nucleotide sequences of 1098 SNPs and silicoDArTs linked to and/or associated with the fertility restoration and nucleotide sequences within a database. The level of alignments between query and subject sequences was determined by the percentage of the query sequence that covered a sequence in Genbank (QC%), percentage of identity over the length of the coverage area (I%), and probability value (E-value). The taxonomic category selected during searches was the *Poaceae* family.

### Marker conversion to PCR-based assays

Markers linked to or/and associated with fertility restoration were converted into PCR-based assay. Initially, due to very short sequences (69-bp), SNPs and silicoDArTs were blasted with rye Lo7 WGS contigs (Bauer et al. 2017; https://webblast.ipk-gatersleben.de/ryeselect/). Lo7 WGS contigs which show 100% sequence homology with the marker sequence, were analyzed in Primer3web software version 4.1.0 (http://bioinfo.ut.ee/primer3/) to identify primer pairs for marker amplification. The primer design’s main criteria were as follows: primer size 18–22 bp, GC content 40–60%, no or negligible secondary structures, and product size ≥ 400 bp. The optimal annealing temperature was inferred using a gradient PCR with temperatures set between 51.0 and 65.0°C (Labcycler Gradient, SensoQuest GmbH). DNA of sterile (S305P/00) and fertile (SO2R/05) parental plants were used to test marker polymorphism. Reaction mixtures had a final volume of 10 μl consisting of 10 ng of total genomic DNA, 25 μM each of PCR primers, 2.5 mM dNTPs, 2.5 mM MgCl2, 1 X reaction buffer, and 0.25 U of Gold HotStart DNA Polymerase (Syngen Biotech Ltd.). The following reaction profile was applied: [95 °C–15′] [95 °C-30″; X°C–45″; 72 °C–45″] × 35 [72 °C–10′] [5 °C ∞], when “X” is the temperature selected based on PCR reaction in gradient profile. Amplification products were separated by electrophoresis at 5 V/cm for 1.5 h in TBE buffer in 1.2% agarose gels containing 0.5 µg/ml of ethidium bromide. PCR-based markers’ segregation profile was tested using DNA of 20 sterile and 20 fertile lines randomly chosen from a set of the S60/08 mapping population. Converted PCR-based markers have extended names with “c” in the end (i.e., 3744672 vs. 3744672c).

## Results

### Phenotyping

The BC1F1: S305P/00 × [RIL F8 (S60/08): S 305 N/00 × SO 2R/05] crosses were characterized by either sterile, partly fertile, or fully fertile values of the trait. According to the established criteria, 58 sterile, 3 partly fertile, and 30 fertile RILs were identified (Supplementary File S1). Phenotypes were not obtained for 3 out of the 94 cases. Statistical analysis showed that the trait does not follow normal distribution (W = 0.780, p value < 0.0001, α = 0.05).

Chi-square adjustment tests revealed that the population deviated significantly from the expected 1:1 sterile-to-fertile segregation ratio (*χ*^2^ = 4090, p < 0.01 at α < 0.05) when contrasting sterile (lines with 1–3 bonitation) and fertile (lines with 4–9 bonitation) phenotypic classes were analyzed.

### Genotyping and genetic map construction

The segregation patterns for 47960 SNP and 165163 silicoDArT markers were tested using DArT-Seq™ technology to genotyping the S60/08 mapping population. After removing markers with monomorphic signals and exhibiting >75% missing data, about 16,100 SNP and 50 200 silicoDArT markers were employed for MultiPoint Ultra-Dense and Tassel software.

The genetic map of RIL F8: [(S60/08): S 305 N/00 × SO 2R/05] consists of seven linkage groups (LG) defined by 35,168 markers (272 skeletons, 2731 redundant, 6837 approximated, and 25,328 redundant to approximated markers) and covers 880.3 cM (Table 1, Figure S1). The most extended LG was constructed for chromosome 2R and spanned over 183.9 cM. The shortest LGs were for the 3R (65.9 cM) and 7R (69.1 cM) ones, with 23 and 36 markers, respectively. On average, one skeleton marker per 3.2 cM was located on the map. The average saturation range from one marker per 1.91 cM (LG-7R) to 4.48 cM (LG-2R).

**Table 1:** Arrangement of mapping data evaluated for the S60/08 mapping population

Chromosome		1R	2R	3R	4R	5R	6R	7R	Total	Average
No. of markers	**Skeleton (S)**	57	41	23	42	30	43	36	272	-
	**Redundant to S**	628	574	366	159	192	178	634	2731	-
	**Approximated (A)**	1366	893	563	1491	430	859	1235	6837	-
	**Redundant to A**	4822	3847	2547	6181	962	1942	5027	25328	-
	**Together**	6873	5355	3499	7873	1614	3022	6932	35168	-
**Map length (cM)**		145.9	183.9	65.9	159.2	85.7	170.6	69.1	880.3	-
**Max gap (cM)**		13.1	22.4	14.8	13.4	15.1	25.1	6.7	-	-
**Saturation (marker/cM)**		2.55	4.48	2.86	3.79	2.85	3.96	1.91	-	**3.20**

### Pollen fertility restoration QTLs

The Kruskal–Wallis (K-W) analysis in the RIL F8: [(S60/08): S 305 N/00 × SO 2R/05] population revealed two, one, thirteen, one, and three significant markers (p ≤ 0.01) mapped to chromosomes 1R, 3R, 4R, 6R, and 7R, respectively. The maximum K-W statistic values for markers mapped to the 1R, 3R, 6R, and 7R chromosomes ranged from 7.4 to 10.6. The most significant markers (p ≤ 0.005) fall in the interval 112.9 cM-136.82 cM (Table 2) of the 4R chromosome. The region corresponds to the 4R QTL responsible for fertility restoration. The association values (K_max_^2^) for those markers were ≥ 9.28, with the highest value (K_max_^2^ = 29.414) for marker 7464414 at the position of 134.85 cM. Moreover markers 7468190 (136.16 cM), 5505157 (136.82 cM), 3744723 (128.85 cM) and 5044143 (130.87 cM) exceeded the K_max_^2^ value of twenty.

**Table 2:** The association between markers and fertility restoration trait in rye with CMS Pampa based on the Kruskal–Wallis test for markers with significance values (p) equal or less than 0.01

Chromosome	Marker position (cM)	Marker name and significance^*1*^	K_max_^*2*^
1R	67.56	100087134****	7.571
1R	68.12	3576049***	7.988
3R	14.49	3364812****	8.714
4R	44.77	3588313****	9.282
4R	112.99	5137834******	14.890
4R	114.99	5488867****	10.127
4R	116.99	3364527*******	19.567
4R	122.15	3735158****	9.293
4R	128.85	3744723*******	22.816
4R	130.87	5044143*******	20.099
4R	131.53	3601185*******	19.155
4R	132.88	3577181*******	27.686
4R	134.85	7464414*******	29.414
4R	136.16	7468190*******	22.837
4R	136.82	5505157*******	20.702
4R	145.86	5205638****	9.226
6R	45.96	100002600***	7.423
7R	56.70	5139199****	8.769
7R	57.87	5497518****	9.167
7R	59.62	100059968****	10.602

^*1*^Significance levels: *p ≤ 0.1, **p ≤ 0.05, ***p ≤ 0.01, ****p ≤ 0.005, *****p ≤ 0.001, ******p ≤ 0.005, *******p ≤ 0.0001

^*2*^K_max_^2^ marker association value

**Fig. 1:**
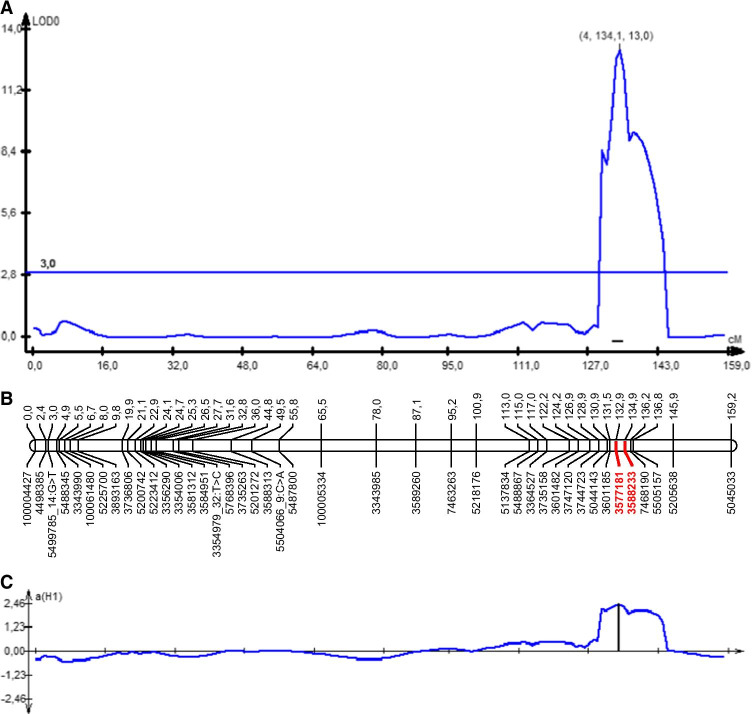
Composite interval mapping (CIM) demonstrating the position (**A**) of the *QRft-4R* identified on the 4R chromosome (**B**) based on the RIL S60/08 mapping population. The main effect detected on chromosome 4R is an additive effect arising in restorer line SO2R/05 (**C**)

Composite interval mapping (CIM) indicated the presence of a QTL conferring fertility restoration in rye with CMS Pampa in the range of 128.85–136.8 cM. A highly significant QTL (*QRfp-4R*) with the LOD score of 10.15 (*p* = 1000; LOD = 3.1) mapped to the distal part of the long arm of the 4R chromosome (Fig. 1, Table 3). The QTL exhibited additive effects (A = 2.14) and explained 42.3% of the phenotypic variance for pollen fertility restoration. The silicoDArT markers 3588233 and 3577181 surrounded the QTL from both sides at a distance of 0.84 cM and 1.13 cM apart from the LOD maximum, respectively (Table 3). Besides, the QTL region was saturated with 25 redundant and 878 approximated markers.

**Table 3:** Composite interval mapping (CIM) outcomes arrangement

Chr	QTL	Closest markers	Number of redundant markers	Distance from QTL LOD^*1*^ maximum position (cM)	LOD maximum position (cM)	LOD value maximum	A^*2*^	R^2^ (%)^*3*^	RecL^*4*^	RecR
4R	QRfp-4R	3588233 3577181	11 3	0.85 1.12	134.00	13.06	2.41	42.3	0.000	0.019

^*1*^LOD – the logarithm of odds

^*2*^A is the value of the additive effect of the SO2R/05 allele

^*3*^*R*^2^ (%) – the percentages of phenotypic variance explained by the given QTL

^*4*^RecL and RecR reflect the value of recombination of the markers in the nearest vicinity of the QTL LOD function maximum

A distance of 1.97 cM separates the markers 3588233 and 3577181 (and their redundant and approximated markers) mapped within the *QRfp-4R*. The marker sequences were identified on a high-density genetic map of rye Lo7xLo225 (Bauer et al. 2017) based on markers-contigs sequence homology. The region ranging from 132.88 to 134.85 cM on the RIL8 map corresponds to the 183.52–188.71 cM region on the Lo7xLo225 map as indicated by 56 approximated and two redundant markers. The markers 3341749 and 100060499, redundant to 3577181 highly linked with *QRfp-4R*, were identified at 183.52 cM and 184.31 cM of the Lo7xLo225 map. The two maps of the 4R chromosome were strongly correlated 0.994 (p < 0.01).

### Association mapping

Principal Coordinate Analysis (PCoA) performed for all RIL F8: [(S60/08): S 305 N/00 × SO 2R/05] lines encompassing mapping population failed to detect significant data structuring (not shown). The GLM allowed identifying 241 SNP and silicoDArT markers associated with the pollen fertility trait at α ≤ 0.01. Most of those markers had redundant counterparts, increasing the total number of associated markers to 1098. The association coefficients (R^2^) of the markers that passed the Bonferroni test’s cut-off value (p = 4.91E − 06) ranged from 0.59 to 0.21 (Table 4). The R^2^ association value of the silicoDArTs closely linked to the *QRfp-4R* reached 0.4 and 0.29 for 3588233 and 3577181, respectively. The markers approximated to these two skeleton markers' position were also associated with the fertility restoration trait and reached R^2^ values from 0.22 to 0.59 (Table S1).

**Table 4:** SNP and silicoDArT markers with the highest association values for the male-fertility restoration in RIL S60/08 mapping population of rye with CMS Pampa

Marker code	Chr	Marker_p	R^2^	*Number of redundant markers
100005393	n.a.^	3.39E − 18	0.59	-
5043774_9:C > T	4R	1.24E − 17	0.56	1
3348274	4R	6.56E − 17	0.53	22
3591087_11:A > G	4R	9.88E − 17	0.49	9
3349009	4R	1.85E − 16	0.57	16
3597371_24:A > C	4R	1.51E − 15	0.55	32
3582094	n.a	1.95E − 15	0.51	5
3576814_28:A > G	4R	4.15E − 15	0.51	2
3584536_28:G > A	4R	8.91E − 15	0.51	3
5037146_7:C > G	4R	3.00E − 15	0.61	1
3586955	4R	1.25E − 14	0.52	3
100105450	n.a	5.97E − 14	0.49	-
100005329	n.a	5.11E − 14	0.49	3
3353442	n.a	5.63E − 14	0.48	-
7468405_22:A > G	n.a	8.98E − 13	0.53	-
3579773	4R	9.96E − 13	0.47	1
3354484	n.a	6.87E − 13	0.49	-
7468019	n.a	4.02E − 13	0.46	3
3732815	4R	7.34E − 13	0.45	11
5500161	4R	2.22E − 12	0.46	-

^*^Based on segregation pattern

^Not assigned

Blasting marker sequences associated with pollen fertility trait in the RIL8 against the Lo7 WGS contigs (Bauer et al. 2017) showed 65 out of 89 markers localized between 179.5 cM and 193.91 cM. Twenty-three markers with the highest association values (≤ E − 10) with the trait were within the range of 183.52–188.71 cM on the 4R chromosome of the Lo7xLo225 map.

### Identifying marker sequence homology with known functional genes

DNA sequences of the markers linked to and/or associated with the pollen fertility restoration QTL assigned on the 4R chromosome were blasted against DNA sequence databases. Of these, 34% of the analyzed sequences showed sequence similarity to genomic sequences from *Triticum aestivum*, *Triticum dicoccoides*, *Hordeum vulgare* subsp. *vulgare*, and *Aegilops tauschii* subsp. *tauschii* (Table S1).

Four of the markers (5221039, 3744672, 5036421, 3749937) associated with the fertility restoration and identified in the *QRfp-4R* region showed sequences homology to the *Rf1* protein-coding sequence from *Triticum dicoccoides* (Zhu et al. 2019)*.* The markers were assigned to the 4R linkage group at 132.8 cM of the S60/08 and 187.9 cM of the Lo7xLo225 reference map (Bauer et al. 2017). Another twenty-six marker sequences (5500712, 3741145, 5227369_10:C > G, 3591947, 3362765, 3344372, 5037650, 3730066, 100062369, 5227369, 3738490, 5227369_7:G > A, 3585416, 5493400, 100089652, 3895196, 3577569_6:C > G, 3896198_10:G > T, 100104448_33:A > C, 3599981_67:C > G, 5504322, 3349918, 3887543_35:C > A, 12699528, 5227367_28:G > A, 3587492) were homologous to the sequence of *Rfm1* restorer locus identified in *Hordeum vulgare* that carries two, three and one regions encoding pentatricopeptide (PPR) repeat-containing protein, putative beta-fructofuranosidase, and putative zinc finger with peptidase domain protein, respectively (Rizzolatti et al. 2017; GenBank: MF443757.1)*.* Six (3349917, 5227368GA7, 5227368GA28, 5227368CG10, 5493409, 5504321) and two marker sequences (3362765, 3738490) were homologous to putative beta-fructofuranosidase and putative zinc finger with peptidase, respectively. The remaining markers were outside of coding regions, and any of the marker sequences matched the region coding PPR protein. Additionally, fifteen markers were mapped in the *QRfp-4R* region of the S60/08 genetic map and eleven in the Lo7xLo225 genetic map (183.51 cM and 185.52 cM).

Nineteen of the marker sequences (3357230, 3601082, 3885888, 7463614, 4489713_14:A > C, 3593839, 3351619, 3580257, 3358064, 3730381, 100089972, 3884378, 3358394, 5487641, 5139216, 3592314_41:C > G, 3576186_29:G > A, 100005251, 3576186) were homeologous to the sequences of transcription termination factor family (mTERF) genes from *Triticum dicoccoides. *Seven of them (3358394, 5487641, 5139216, 3592314_41:C > G, 3576186_29:G > A, 100005251, 3576186) were homologous to the MTERF8 gene (E-value from 1e − 13 to 1e − 27; Query cover 94–100%) and mapped at 180.33 cM position of the reference map, corresponding to the position of Lo7_v2_contig_62134. The markers covered the 23546 bp-long sequence of Lo7_v2_contig_62134 between 15415 bp and 15894 bp. Another seven marker sequences homologous to MTERF15 were identical or highly similar (E-value from 8e − 16 to 3e − 27; query cover 99–100%) to the nucleotide sequences of Lo7_v2_contig_4941 (3885888, 3593837, 3601082), Lo7_v2_contig_2489 (7463614, 3351619) and Lo7_v2_contig_4048 (3580257, 3357230) localized at the positions of 185.93 cM, 185.93 cM, and 186.72 cM of Lo7xLo225 genetic map, respectively.

Eight markers with one of the highest association values (3348274, 3358169, 3746061, 4096992, 3590786, 5037479, 7468019, 3586369_11:C > G) exhibited sequence similarity to the DNA sequence of the keratin-associated protein (KAP) 5–4-like and 5–5-like genes from *Aegilops tauschii.*

### Conversion of SNP and silicoDArT markers to PCR-based assays

There were 38 markers linked to *QRfp-4R* and/or associated with fertility restoration selected for conversion to specific PCR conditions. The markers were segregated as their unconverted counterparts in ten cases and amplified fragments of expected sizes in the range of 363–632 bp (Table 5). The remaining cases resulted in segregation distinct from their SNP or silicoDArT counterparts (5 markers) or were monomorphic. The efficiency of marker conversion equaled 26.3%.

**Table 5:** Primer sequences and PCR conditions for markers linked to or/and associated with fertility restoration in rye with CMS Pampa

Marker name	Sequences (5′ – > 3′) *	Tm (°C)	Product size (bp)	Sequence homology (BLAST)
3744672c	F: gtggcttctcttctcgtt	55.0	363	PREDICTED: *Triticum dicoccoides* protein Rf1
	R: atcaactccctcctcacc			
3599981c	F: gtatagtgcagagaggag	49.0	445	*Hordeum vulgare* subsp. *vulgare* cultivar Morex Rfm1 gene locus, complete sequence
	R: agcaaccatccaaggaac			
5500712c	F: accaaggctgctacaaagga	62.5	615	*Hordeum vulgare* subsp. *vulgare* cultivar Morex Rfm1 gene locus, complete sequence
	R: tcgtggcaacctctcttaca			
3362765c	F: gatcctctccctcgcaatcc	57.0	417	*Hordeum vulgare* subsp. *vulgare* cultivar Morex Rfm1 gene locus, partial sequence
	R: tctgaacctcgtcatcctcg			
3358169c	F: gtgctggtgtttgtggtgtt	63.5	423	PREDICTED: *Aegilops tauschii* subsp. *tauschii* keratin-associated protein 5–4-like
	R: tcaggcggtgacacagtt			
7468190c	F: tcatcagccgtgtgtagtgt	60.4	590	PREDICTED: *Aegilops tauschii* subsp. *tauschii* cytochrome P450 709B2-like
	R: cgtcgcacatctagttgcaa			
3593839c	F: agccgcagtttctacctcat	57.0	531	PREDICTED: *Triticum dicoccoides* transcription termination factor MTERF15
	R: aaattagctggcacttcccc			
4099883c	F: cttgcctgcacttgaagagg	62.5	626	-
	R: gcgctacagaaaactgcact			
3575914c	F: ttcggggagagcatgatacc	62.5	632	-
	R: cacaaatcgaaggggagcag			
3602675c	F: acagggatcaaaggggtcag	62.5	487	-
	R: acacatgtacagcccgagaa			

F: forward; R: reverse

^*^Primer information is available for non-commercial use on request by prof. Piotr Bednarek, Plant Breeding and Acclimatization Institute – NRI, 05–870 Błonie, Radzików, Poland

Four of the successfully converted markers show sequence homology with the *Rf1* protein-coding sequence (3744672c) and the sequence of *Rfm1* gene locus (5500712c, 3599981c, 3362765c) responsible for fertility restoration in *Triticum dicoccoides* and *Hordeum vulgare*, respectively. The 5500712c, 3599981c localized in the non-coding region of *Rfm1*, while primers based on 3362765 marker sequence amplified 417 bp fragment of the putative zinc finger with peptidase domain protein. One primer pair (Table 5) based on 3593839c marker sequence amplified the 531 bp region of the MTERF15 gene sequence. The amplified region of MTERF15 encompasses sequences complementary to four marker sequences (3593839, 3601082, 4489713_14:A > C, 3351619). Detailed results are presented in Table 5.

## Discussion

The phenotype analysis of the cross S305P/00 x [RIL F8 (S60/08): S 305 N/00 × SO 2R/05] classified most BC1F1 crosses into two contrasting classes: entirely male sterile and fully male fertile. Most of the RILs exhibited sterile, whereas fully fertile plants were represented by 33% of the RILs. Interestingly, only three lines were classified as partially fertile. It was also noted that the trait failed to follow a normal distribution. Furthermore, the segregation ratio deviated from the monogenic mode of segregation, indicating that more than a single gene may participate in pollen restoration.

On the other hand, the presence of sterile and fertile RILs in the population and practically lack of partial phenotypes may suggest that a single QTL may act as a dominant one in the presence of some weak QTLs conferring sterility. Although phenotyping was conducted in a single environment, we did not expect significant errors related to the incorrect assessment of plants' fertility/sterility. It was documented that pollen fertility restoration in rye with CMS Pampa determined by QTL with significant effects on the trait’s phenotypic variation is less affected by environmental conditions (Geiger et al. 1995). The high correlation of the phenotyping results in two environments was obtained by Stracke et al. (2003) (r = 0.94 and r = 0.92 for mapping populations included 651 and 498 individuals, respectively) and Miedaner et al. (2000) (r ≥ 0.9 for three F2 populations of 131, 134, and 100 individuals). Genetic map construction is necessary to verify the number of QTLs responsible for pollen fertility restoration in our RIL8 mapping population.

We have successfully applied SNP and silicoDArT markers for genotyping RIL-based mapping populations of rye (Niedziela et al. 2021) and triticale (Wasiak et al. 2021). The NGS markers proved to efficient in identifying many markers that were more or less evenly distributed along species chromosomes saturated maps with 1.66 (rye) and 1.70 (triticale) markers per every one cM (Niedziela et al. 2021; Wasiak et al. 2021). Preliminary, the S60/08 mapping population encompassed 130 lines, but due to the inbreeding depression, a phenomenon typical for allogamous species such as rye (Husband and Schemske 1996), 30 lines of mapping population was lost. As a result, map saturation was lower than in our previous study, whereas many as 175 RILs were used (Niedziela et al. 2021). Still, all linkage groups' marker saturation was sufficient for quantitative analysis, possibly due to many recombinations typical for such populations (Xu et al. 2017). However, some gaps ranging from 6.7 cM and 25.1 cM were not eliminated, but their sizes in most cases were limited. The gaps were present close to centromeric regions, which is quite typical in genetic mapping as these regions recombine with lower frequency (Stapley et al. 2017).

A single marker analysis (SMA) approach was applied to identify genomic regions responsible for pollen fertility restoration present in the S60/08 mapping population. The analysis indicates that the trait might be conferred by regions mapped to the 1R, 3R, 4R, 6R, and 7R chromosomes. However, the central region, represented by the prevailing number of markers conferring ca. 8 cM was present on the 4R chromosome. The result agrees with our phenotypic data suggesting the presence of a single region conferring pollen fertility and some set of other, less critical genes. The presented data are in line with results presented by the others where the same chromosomes conferring the trait were identified (Miedaner et al. 2000).

Furthermore, the biparental population has a significant genomic region on the 4R, which is not typical for the European rye population (Miedaner et al. 2000; Hackauf et al. 2017). The presented data is congruent with composite interval mapping. The QTL explaining the vast part of the trait’s phenotypic variance (42.3%), exhibiting additive effect, was identified on chromosome 4R. The CIM and SMA approach indicated the same genomic region of the chromosome. It should be stressed that for non-normally distributed traits, CIM is not an adequate approach (Broman 2003). However, in the case of pollen fertility, the approach works well and gives comparable results with SMA (Myśków et al. 2014; Stojałowski et al. 2017; Masojć et al. 2020). The markers tightly linked to QTL were either in the QTL maximum (RecL) or 0.02 cM (RecR) apart from the QTL maximum.

Furthermore, CIM (and SMA) showed that the QTL most significant region spans over ca. 2 cM. It was possible to detect such a narrow region thanks to the advanced recombination inbred line-based mapping population. Interesting, the markers covering the 4R chromosome, including those linked to the *QRfp-4R*, were in the same order in the case of the Lo7xLo225 population (Bauer et al. 2017), supporting congruency of the presented data. Both SMA and CIM succeeded in identifying ca. 1960 markers in the *QRfp-4R* region between 128.85 and 136.8 cM that could be utilized for marker conversion and then used for marker-assisted selection purposes.

The approach used for identifying markers linked to the trait of interests may result in eliminating some valuable markers due to marker segregation problems or missing data (Ronin et al. 2015). In the current study, as many as 70% of markers were omitted. The very similar results were presented by the others (Bolibok-Brągoszewska et al. 2009; Milczarski et al. 2011; Stojałowski et al. 2017). These drawbacks could be overcome by association mapping. Biparental mapping populations usually should lack any population structure (Che and Xu 2012; Stadlmeier et al. 2018), allowing for implementing the general likelihood method for identifying associated markers. The approach proved to be valuable in the S60/08 mapping population, allowing for recognizing extra 22 markers with association values close to 0.6. The vast majority of the significant markers with the highest values of association with pollen fertility were directly identified in the S60/08 as redundant or approximated markers or were present within the respective region in the case of the Lo7xLo225 reference genetic map (Bauer et al. 2017).

An essential aspect of the study was the opportunity to identify genes involved in pollen fertility restoration in rye with CMS Pampa. Twenty-six marker sequences localized within the *QRfp-4R* region revealed sequence homology with the sequence of *Rfm1* locus responsible for the fertility restoration in barley with *male sterility maternal 1* (*msm1*) sterile cytoplasm (Matsui et al. 2001) and carries, among others, two tandem repeat genes coding for pentatricopeptide repeat (PPR) protein (Rizzolatti et al. 2017). Previous synteny-based studies showed the homology between the barley 6HS chromosome region (Martis et al. 2013) carrying *Rfm1* locus and 4RL of rye where the *Rfp1* and *Rfp3* genes were mapped (Hackauf et al. 2017). However, a slight shift of *Rfp1* and *Rfp3* to the *Rfm1* locus (Hackauf et al. 2012, 2017) suggests that the CMS systems in barley and rye base on tightly linked but independent restorer genes or the shift results from local duplication of the same restorer gene (Rizzolatti et al. 2017). It was documented that *Rfm1*-orthologous regions of *Brachypodium* (Bradi3g00900) and rice (Os02g0106300) encodes PPR proteins, while the *Rfp1* syntenic interval present in those two species does not harbor any PPR genes (Rizzolatti et al. 2017; Hackauf et al. 2017). The precise position of silicoDArT and SNP sequences within the *Rfm1* (GenBank: MF443757.1) showed that any of them matched the region coding PPRs. Nevertheless, nine of the marker sequences (3895196; 100104448_33:A > C; 3896198_10:G > T; 5227369_7:G > A, 5227369_28:G > A, 5227369_10:C > G; 5493400; 5504322; 3349918) matched three regions coding putative beta-fructofuranosidase (proteins id.: AVY91566.1, AVY91567.1, and AVY91565.1) whereas two other marker sequences (3362765; 3738490) were homologous to the region coding putative zinc finger with peptidase domain protein (protein id. AVY91564.1).

The mTERF proteins are the second vital candidates for fertility restoration in cereals. Genome-wide association studies in a multiparental mapping population of barley revealed three markers located very close to the mTERF gene or lying directly within the mTERF gene sequence from the *Rfm3* restorer locus (Bernhard et al. 2019). In wheat with male sterility induced by the cytoplasm of *Triticum timopheevii*, the genes encoding mTERF and PPR proteins were detected within *the Rf9* locus on chromosome 6AS (Shahinnia et al. 2020). The candidate genes coding for mTERF proteins located between the flanking markers of the *Rf9* in wheat was highly expressed in the spikes and grains. Six additional, tandemly duplicated genes predicted to encode for mTERF proteins are located on rice chromosome 6 within a region orthologous to the rye *Rfp1* (Hackauf et al. 2012). Nineteen of the markers exhibiting sequence similarity to the sequence of the mTERF family were present within *QRfp-4R*. There were approximated to the S60/08 map mainly at the positions 132.88 cM (7 markers) and 134.85 cM (6 markers). Further bioinformatic analysis indicated two possible positions of mTERF on the rye genetic map based on the Lo7xLo225 mapping population (180.33 cM and 185.93 cM) (Bauer et al. 2017). One of the silicoDArT (3593839c) converted into PCR condition assay showed polymorphic segregation compatible with the segregation before conversion. Surprisingly, one of the highest association values (R^2^ = 0.53) was obtained for markers that exhibited similarity to the keratin-associated protein’s DNA sequence (KAP) 5–4-like and 5–5-like from *Aegilops tauschii* subsp. *tauschii*. So far, the role of those proteins in plants has been poorly understood. The expression study results in maize suggested that KAP5-4 participated in response to water-deficit stress (Yang et al. 2012). BLAST analysis show homology (54.84%) of maize KAP5-4 with the qPE9-1 encoding KAP5-5 in rice. The qPE9-1, which is allelic to DEP1 (DENSE AND ERECT PANICLE 1), plays an integral role in the regulation of rice plant architecture, including panicle erectness (Zhou et al. 2009), and it is a positive regulator of rice grain length and weight (Li et al. 2019). Nevertheless, comparative study of KAP 5–4 and 5–5 protein sequences of maize and rice do not indicate significant homology with those proteins for *Aegilops tauschii* subsp. *tauschii.*

Besides scientific interest, the current study has practical application. We succeeded in converting some of our linked/associated markers to PCR-specific conditions with nearly 30% of success typical for other plant species (Fiust et al. 2015; Niedziela et al. 2015). In the current study, we used marker sequences to identify their homologous sequences in databases. The respective database sequences were used to design primers amplifying DNA fragments ranging from 363 to 632 bp. Unfortunately, many of the amplified fragments were monomorphic, probably because we failed to amplify polymorphic regions. Some markers had segregation patterns that did not follow the pattern of the original markers. Such a situation is most probably related to copies of the amplified sequence, which is not surprising in the rye (Guidet et al. 1991; Bauer et al. 2017).

In conclusion, the highly significant QTL responsible for fertility restoration in line SO2R/05 with CMS Pampa (restorer line) mapped to the distal part of the 4R chromosome. A group of silicoDArT and SNP markers was tightly linked to the *QRfp-4R* and/or associated with the trait. Successful conversion led to obtaining ten markers potentially applicable in commercial breeding. There were 5500712c, 3599981c, 3362765c, and 3744672c markers that revealed sequence homology with the sequences of fertility restoration genes identified in the other cereals. The markers can be helpful as a tool for further genetic analyses of the CMS-Pampa system and the QTL selection on the 4R chromosome.

## Supplementary Information

Below is the link to the electronic supplementary material.

Supplementary file1 (DOCX 63 KB)

Supplementary file2 (XLSX 54 KB)

Supplementary file3 (XLSX 26 KB)

## References

[CR1] Adolf K, Winkel A (1985) A new source of spontaneous sterility in winter rye – preliminary results. Proc. Eucarpia Meet. Cereal Sect. Rye, Svalöv, Sweden, 11–13.06, pp 293–306

[CR2] Akagi H, Nakamura A, Yokozeki-Misono Y, Inagaki A, Takahashi H, Mori K, Fujimura T (2004). Positional cloning of the rice Rf-1 gene, a restorer of BT-type cytoplasmic male sterility that encodes a mitochondria-targeting PPR protein. Theor Appl Genet.

[CR3] Bauer E, Schmutzer T, Barilar I, Mascher M, Gundlach H, Martis MM, Twardziok SO, Hackauf B, Gordillo A, Wilde P, Schmidt M, Korzun V, Mayer KFX, Schmid K, Schön C-C, Scholz U (2017). Towards a whole-genome sequence for rye (*Secale cereale* L). Plant J.

[CR4] Bernhard T, Koch M, Snowdon RJ, Friedt W, Wittkop B (2019). Undesired fertility restoration in *msm1* barley associates with two mTERF genes. Theor Appl Genet.

[CR5] Bolibok-Brągoszewska H, Heller-Uszyńska K, Wenzl P, Uszyński G, Kilian A, Rakoczy-Trojanowska M (2009). DArT markers for the rye genome - genetic diversity and mapping. BMC Genomics.

[CR6] Bradbury PJ, Zhang DE, Kroon TM, Casstevens Y, Ramdoss Y, Buckler ES (2007). TASSEL: software for association mapping of complex traits in diverse samples. Bioinformatics.

[CR7] Broman KW (2003). Mapping quantitative trait loci in the case of a spike in the phenotype distribution. Genetics.

[CR8] Brown GG, Formanová N, Jin H, Wargachuk R, Dendy C, Patil P, Laforest M, Zhang J, Cheung WY, Landry BS (2003). The radish Rfo restorer gene of Ogura cytoplasmic male sterility encodes a protein with multiple pentatricopeptide repeats. Plant J.

[CR9] Che X, Xu S (2012). Generalized linear mixed models for mapping multiple quantitative trait loci. Heredity (edinb).

[CR10] Dahan J, Mireau H (2013). The Rf and Rf-like PPR in higher plants, a fast-evolving subclass of PPR genes. RNA Biol.

[CR11] Desloire S, Gherbi H, Laloui W, Marhadour S, Clouet V, Cattolico L, Falentin C, Giancola S, Renard M, Budar F, Small I, Caboche M, Delourme R, Bendahmane A (2003). Identification of the fertility restoration locus, Rfo, in radish, as a member of the pentatricopeptide-repeat protein family. EMBO Rep.

[CR12] Fiust A, Rapacz M, Wójcik-Jagła M, Tyrka M (2015). Development of DArT-based PCR markers for selecting drought-tolerant spring barley. J Appl Genet.

[CR13] Fujii S, Kazama T, Ito Y, Kojima S, Toriyama K (2014). A candidate factor that interacts with RF2, a restorer of fertility of Lead rice-type cytoplasmic male sterility in rice. Rice.

[CR14] Fujii S, Suzuki T, Giegé P, Higashiyama T, Koizuka N, Shikanai T (2016). The Restorer-of-fertility-like 2 pentatricopeptide repeat protein and RNase P are required for the processing of mitochondrial orf291 RNA in *Arabidopsis*. Plant J.

[CR15] Geiger HH, Miedaner T (1996). Genetic basis and phenotypic stability of male-fertility restoration in rye. Vortr Pflanzenzüchtg.

[CR16] Geiger HH, Morgenstern K (1975). Angewandt-genetische Studien zur cytoplasmatischen Pollensterilität bei Winterroggen. Theor Appl Genet.

[CR17] Geiger HH, Schnell FW (1970). Cytoplasmic male sterility in rye (*Secale cereale* L.). Crop Sci.

[CR18] Geiger HH, Yuan Y, Miedaner T, Wilde P (1995) Environmental sensitivity of cytoplasmic genic male sterility (CMS) in *Secale cereale* L. In: Kück U, Wricke G (eds) Genetic mechanisms for hybrid breeding. Adv Plant Breed 18:7–17

[CR19] Goryunov DV, Anisimova IN, Gavrilova VA, Chernova AI, Sotnikova EA, Martynova EU, Boldyrev SV, Ayupova AF, Gubaev RF, Mazin PV, Gurchenko EA, Shumskiy AA, Petrova DA, Garkusha SV, Mukhina ZM, Benko NI, Demurin YN, Khaitovich PE, Goryunova SV (2019). Association mapping of fertility restorer gene for CMS PET1 in sunflower. Agronomy.

[CR20] Guidet F, Rogowsky P, Taylor C, Song W, Langridge P (1991). Cloning and characterisation of a new rye-specific repeated sequence. Genome.

[CR21] Hackauf B, Bauer E, Korzun V, Miedaner T (2017). Fine mapping of the restorer gene Rfp3 from an Iranian primitive rye (*Secale**cereale* L.). Theor Appl Genet.

[CR22] Hackauf B, Korzun V, Wortmann H, Wilde P, Wehling P (2012). Development of conserved ortholog set markers linked to the restorer gene Rfp1 in rye. Mol Breed.

[CR23] Hammani K, Giegé P (2014). RNA metabolism in plant mitochondria. Trends Plant Sci.

[CR24] Hansen HB, Moller B, Andersen SB, Jorgensen JR, Hansen A (2004). Grain characteristics, chemical composition, and functional properties of rye (*Secale cereale* L.) as influenced by genotype and harvest year. J Agric Food Chem.

[CR25] Husband BC, Schemske DW (1996). Evolution of the magnitude and timing of inbreeding depression in plants. Evolution.

[CR26] Jaccoud D, Peng K, Feinstein D, Kilian A (2001). Diversity arrays: a solid state technology for sequence information independent genotyping. Nucleic Acids Res.

[CR27] Kilian A, Wenzl P, Huttner E, Carling J, Xia L, Blois H, Caig V, Heller-Uszynska K, Jaccoud D, Hopper C, Aschenbrenner-Kilian M, Evers M, Peng K, Cayla C, Hok P, Uszynski G, Pompanon F, Bonin A (2012). Diversity Arrays Technology: A Generic Genome Profiling Technology on Open Platforms. Data Production and Analysis in Population Genomics. Methods in Molecular Biology (Methods and Protocols).

[CR28] Klein RR, Klein PE, Mullet JE, Minx P, Rooney WL, Schertz KF (2005). Fertility restorer locus Rf1 of sorghum (*Sorghum bicolor* L.) encodes a pentatricopeptide repeat protein not present in the colinear region of rice chromosome 12. Theor Appl Genet.

[CR29] Kobyljanskij VD (1969). About genetics of cytoplasmic male sterility in winter rye (K genetike citoplazmatičeskoj mužskoj steril’nosti u ozimoj rži). Genet Moskva.

[CR30] Kubo T, Arakawa T, Honma Y, Kitazaki K (2020). What does the molecular genetics of different types of restorer-of-fertility genes imply?. Plants.

[CR31] Laidig F, Piepho HP, Rentel D, Drobek T, Meyer U, Huesken A (2017). Breeding progress, variation, and correlation of grain and quality traits in winter rye hybrid and population varieties and national on-farm progress in Germany over 26 years. Theor Appl Genet.

[CR32] Li X, Tao Q, Miao J, Yang Z, Gu M, Liang G, Zhou Y (2019). Evaluation of differential qPE9-1/DEP1 protein domains in rice grain length and weight variation. Rice (NY).

[CR33] Lurin C, Andrés C, Aubourg S, Bellaoui M, Bitton F, Bruyère C, Caboche M, Debast C, Gualberto J, Hoffmann B, Lecharny A, Le Ret M, Martin-Magniette ML, Mireau H, Peeters N, Renou JP, Szurek B, Taconnat L, Small I (2004). Genome-wide analysis of *Arabidopsis* pentatricopeptide repeat proteins reveals their essential role in organelle biogenesis. Plant Cell.

[CR34] Łapiński M (1972). Cytoplasmic-genic type of male sterility in *Secale montanum* Guss. Wheat Inform Serv.

[CR35] Łapiński M, Stojałowski S (2003). Occurrence and genetic identity of male sterility-inducing cytoplasm in rye (*Secale* spp.). Plant Breed Seed Sci.

[CR36] Martis MM, Zhou R, Haseneyer G, Schmutzer T, Vrána J, Kubaláková M, König S, Kugler KG, Scholz U, Hackauf B, Korzun V, Schön CC, Doležel J, Bauer E, Mayer KFX, Stein N (2013). Reticulate evolution of the rye genome. Plant Cell.

[CR37] Masojć P, Kruszona P, Bienias A, Milczarski P (2020). A complex network of QTL for thousand-kernel weight in the rye genome. J Appl Genetics.

[CR38] Matsui K, Mano Y, Taketa S, Kawada N, Komatsuda T (2001). Molecular mapping of a fertility restoration locus (Rfm1) for cytoplasmic male sterility in barley (Hordeum vulgare L.). Theor Appl Genet.

[CR39] Melonek J, Zhou R, Bayer PE, Edwards D, Stein N, Small I (2019). High intraspecific diversity of restorer-of-fertility-like genes in barley. Plant J.

[CR40] Melville J, Haines ML, Boysen K, Hodkinson L, Kilian A, Smith Date KL, Potvin DA, Parris KM (2017). Identifying hybridization and admixture using SNPs: application of the DArTseq platform in phylogeographic research on vertebrates. R Soc Open Sci.

[CR41] Melz G, Adolf K (1991). Genetic analysis of rye (*Secale cereal* L.). genetics of male sterility of the G-type. Theor Appl Genet.

[CR42] Melz G, Melz G, Hartmann F (2001) Genetics of a malesterile rye of “G-type” with results of the first F1 hybrids. In proc Int Symp on Rye Breed and Gen EUCARPIA, Radzikow, pp 43–50

[CR43] Miedaner T, Glass C, Dreyer F, Wilde P, Wortmann H, Geiger HH (2000). Mapping of genes for male-fertility restoration in ‘Pampa’ CMS winter rye (*Secale cereale* L.). Theor Appl Genet.

[CR44] Miedaner T, Herter CP, Goßlau H, Wilde P, Hackauf B (2017). Correlated effects of exotic pollen-fertility restorer genes on agronomic and quality traits of hybrid rye. Plant Breed.

[CR45] Milczarski P, Bolibok-Brągoszewska H, Myśków B, Stojałowski S, Heller-Uszyńska K, Góralska M, Brągoszewski P, Uszyński G, Kilian A, Rakoczy-Trojanowska M (2011). A high density consensus map of rye (*Secale**cereale* L.) based on DArT markers. PLoS One.

[CR46] Myśków B, Hanek M, Banek-Tabor A, Maciorowski R, Stojałowski S (2014). The application of high-density genetic maps of rye for the detection of QTLs controlling morphological traits. J Appl Genet.

[CR47] Niedziela A, Brukwiński W, Bednarek PT (2021). Genetic mapping of pollen fertility restoration QTLs in rye (*Secale**cereale* L.) with CMS Pampa. J Appl Genet.

[CR48] Niedziela A, Mańkowski D, Bednarek PT (2015). Diversity arrays technology-based PCR markers for marker assisted selection of aluminum tolerance in triticale (x *Triticosecale* Wittmack). Mol Breed.

[CR49] Rizzolatti C, Bury P, Tatara E, Pin PA, Rodde N, Berges H, Budar F, Mireau H, Gielen J (2017) Map-based cloning of the fertility restoration locus *Rfm1* in cultivated barley (*Hordeum vulgare*). Euphytica 213:276. 10.1007/s10681-017-2056-4

[CR50] Ronin Y, Minkov D, Mester D, Akhunov E, Korol A (2015) Building ultra-dense genetic maps in the presence of genotyping errors and missing data. Advances in wheat genetics: from genome to field. Springer, Tokyo, pp 127–133

[CR51] Shahinnia F, Geyer M, Block A, Mohler V, Hartl L (2020). Identification of Rf9, a gene contributing to the genetic complexity of fertility restoration in hybrid wheat. Front Plant Sci.

[CR52] Stadlmeier M, Hartl L, Mohler V (2018). Usefulness of a multiparent advanced generation intercross population with a greatly reduced mating design for genetic studies in winter wheat. Front Plant Sci.

[CR53] Stapley J, Feulner PGD, Johnston SE, Santure AW, Smadja CM (2017) Variation in recombination frequency and distribution across eukaryotes: patterns and processes. Phil Trans R Soc: B3722016045520160455.10.1098/rstb.2016.0455 10.1098/rstb.2016.0455 PMC5698618 29109219

[CR54] Stojałowski S, Hanek M, Orłowska M, Sobczak M (2017). DArT markers linked with genes controlling restoration of male fertility in hybrid rye cultivars with improved pollen shedding. Folia Pomer Univ Technol Stetin Agric Aliment Pisc Zootech.

[CR55] Stracke S, Schilling AG, Förster J, Weiss C, Glass C, Miedaner T, Geiger HH (2003). Development of PCR-based markers linked to dominant genes for male-fertility restoration in Pampa CMS of rye (*Secale cereale* L.). Theor Appl Genet.

[CR56] TIBCO Software Inc. (2017) Statistica (data analysis software system), version 13. http://statistica.io

[CR57] Van Ooijen JW (2004) MapQTL®5, software for the mapping of quantitative trait loci in experimental populations. Kyazma B.V, Wageningen, Netherlands

[CR58] Wang S, Basten C, Zeng Z (2007) Windows QTL Cartographer 2.5. Department of Statistics, North Carolina State University, Raleigh, NC. http://statgen.ncsu.edu/qtlcart/WQTLCart.htm

[CR59] Wang ZH, Zou YJ, Li XY, Zhang QY, Chen L, Wu H, Su DH, Chen YL, Guo JX, Luo D, Long YM, Zhong Y, Liu YG (2006). Cytoplasmic male sterility of rice with boro II cytoplasm is caused by a cytotoxic peptide and is restored by two related PPR motif genes via distinct modes of mRNA silencing. Plant Cell.

[CR60] Wasiak M, Niedziela A, Woś H, Pojmaj M, Bednarek PT (2021). Genetic mapping of male sterility and pollen fertility QTLs in triticale with sterilizing *Triticum timopheevii* cytoplasm. J Appl Genet.

[CR61] XlStat (2019) https://www.xlstat.com/en/solutions/pre-mium. p Accessed 10 December 2018

[CR62] Xu Y, Li P, Yang Z, Xu C (2017). Genetic mapping of quantitative trait loci in crops. Crop J.

[CR63] Yang L, Fu F-L, Deng L-Q, Zhou S-F, Yong T-M, Li W-C (2012). Cloning and characterization of functional keratin associated protein 5–4 gene in maize. Afr J Biotechnol.

[CR64] Zhao N, Wang Y, Hua J (2018). Genome wide identification of PPR gene family and prediction analysis on restorer gene in *Gossypium*. J Genet.

[CR65] Zhou Y, Zhu J, Li Z, Yi C, Liu J, Zhang H, Tang S, Gu M, Liang G (2009). Deletion in a Quantitative trait gene qPE9-1 associated with panicle erectness improves plant architecture during rice domestication. Genetics.

[CR66] Zhu T, Wang L, Rodriguez J, Deal KR, Avni R, Distelfeld A, McGuire PE, Dvorak J, Luo MC (2019). Improved genome sequence of wild emmer wheat Zavitan with the aid of optical maps. G3 (Bethesda).

