# Supplementary material for: New PCR-specific markers for pollen fertility restoration QRfp-4R in rye (Secale cereale L.) with Pampa sterilizing cytoplasm
Source: J Appl Genet. 2021 Jun 25;62(4):545–57. doi: 10.1007/s13353-021-00646-z (PMC8571214; doi:10.1007/s13353-021-00646-z)
Supplement: Supplementary file 1 — Supplementary file1 (DOCX 63 KB) [file 13353_2021_646_MOESM1_ESM.docx]

**Figure S1.** Genetic map of rye based on the RIL S60/08 mapping population.
